# Occurrence and molecular characterization of *Escherichia coli* strains isolated from black grouse (*Lyrurus tetrix*) from the Karkonosze National Park in Poland

**DOI:** 10.1186/s12917-024-03886-3

**Published:** 2024-01-31

**Authors:** Natalia Kwaśna, Maja Majewska, Magdalena Karwańska, Magdalena Siedlecka, Artur Pałucki, Tomasz Piasecki

**Affiliations:** 1 Department of Epizootiology with Exotic Animal and Bird Clinic, Wrocław University of Environmental and Life Sciences, Grunwaldzki Sq. 45, Wrocław, 50-366 Poland; 2 Karkonosze National Park, Chałubińskiego Str. 23, Jelenia Góra, 58-570 Poland

**Keywords:** Antibiotic resistance, Black grouse, *Escherichia coli*, Virulence genes, Wild birds

## Abstract

The purpose of this study was to characterize *Escherichia coli* (*E. coli*) strains isolated from wild black grouse (*Lyrurus tetrix*), carried out due to the crossing of hiking trails with wild bird habitats from the Karkonosze National Park. Twenty-seven *E. coli* isolates were obtained from fecal samples collected during the winter months of 2017 and 2018. The strains were assigned to their relevant phylo-groups and the prevalence of virulence genes characteristic of APEC strains (*irp2*, *astA*, *iss*, *iucD*, *papC*, *tsh*, *vat*, *cva/cvi*, *stx2f*) was checked using PCR analysis. In addition, the phenotypic and genotypic resistance to antibiotics was determined. The entire study provided a better understanding of the potential bacteriological threat to wild birds of the Karkonosze National Park. The results showed that 55.6% of the strains belonged to phylo-group B1 (15/27), 33.3% to group B2 (9/27) and 11.1% to group D (3/27). Among the virulence genes tested, *irp2* was detected in 25.9% of isolates (7/27), *vat* in 22.2% (6/27) and *iucD* in 3.7% (1/27). The tested *E. coli* strains showed susceptibility to most antimicrobials, only 14 (51.9%) of them were intermediate resistant or resistant to sulfamethoxazole. The presence of none of the tested genes responsible for resistance to selected antibiotics was identified. Our research indicates a low level of transfer of antimicrobial substances to the natural environment and confirms the effectiveness of the Karkonosze National Park’s activities to protect and restore black grouse habitats.

**Supplementary Information:**

The online version contains supplementary material available at 10.1186/s12917-024-03886-3.

## Background

*Escherichia coli* (*E. coli*) is a Gram-negative, rod-shaped bacteria belonging to the *Enterobacteriaceae* family, that is commonly found in both humans and animals (mammals and birds) as either an intestinal commensal or a pathogen [1]. Bacteria may transmit among different host species and exchange genes codifying antibiotic-resistance mechanisms are generally thought. The variety of virulence factors produced, as well as their acting mechanisms, makes it possible to distinguish each serotype and allows intraspecies classification of strains. There is a division to intestinal pathogenic strains and those that cause diseases outside the gastrointestinal tract, depending on the location of the infection occurrence [2].

A special group of extraintestinal *E. coli* strains are APEC (Avian Pathogenic *Escherichia coli*), which are a component of the natural microflora of the gastrointestinal tract of birds, but they can cause opportunistic infections in the case of the host immunosuppression [3]. APEC strains are involved in zoonotic transmission, which may be an important etiological factor and a reservoir of zoonoses, due to their genetic similarity to human strains causing urinary tract infections and meningitis such as UPEC (Uropathogenic *E. coli*) and NMEC (Neonatal Meningitis *E. coli*) [4].

*Escherichia coli* is responsible for both posing a public health risk [5] and for many economic losses in the industry of various livestock species [6, 7]. The declining efficiency of antimicrobials, correlated with the increasing antibiotic resistance of microorganisms, concerns that the available treatment options for infections caused by bacteria, including *E. coli*, will be significantly limited.

Wild birds can be a reservoir of antibiotic-resistant bacteria due to the fact that the habitats of these animals are intertwined with human settlements and tourist routes [8, 9]. Moreover, birds may play an important role in the dissemination of microorganisms during migration [10]. The fact that antibiotics enter the environment, e.g. into terrestrial waters, also poses a threat of acquiring drug resistance by bacteria [11]. Despite growing interest in studying the occurrence and pathogenicity of bacterial strains isolated from wild birds, there is still little data on their prevalence, transmission routes, and drug resistance.

The black grouse (*Lyrurus tetrix*) is a widespread species in many countries in Europe and Asia [12]. In Poland, it is a protected bird, found in the south-eastern parts of the country, mainly in wetlands. The population is declining as a result of adverse human activities, climate change, and predation by wild mammals [13]. In 2017, the State Forests launched a programme to restore the wild population of black grouse, which includes the construction of centres through which young reared birds are released into the wild [14]. Ongoing reintroduction programmes may pose the risk of introducing birds that have already come into contact with human-applied therapeutics [15]. This wild bird species, despite its limited population, may be a potential reservoir of antimicrobial-resistant microorganisms [16].

The aim of the study was the molecular characterization of *Escherichia coli* strains isolated from the feces of black grouse (*Lyrurus tetrix)*, birds belonging to the population that inhabits the Karkonosze National Park in Poland. The phylogenetic relationship and origin of the strains - anthropogenic or zoogenic - were determined, and the virulence genes, phenotypic and genotypic sensitivity to selected antimicrobial agents were specified. In addition, this study allows to establish the risk of potential infection, caused by virulent strains of *E. coli*, for the declining black grouse population.

## Results

Overall, *E. coli* strains were isolated from 13,7% (27/197) of the tested fecal samples. All *E. coli* isolates were positive for the *phoA* and *16 S* rRNA genes, which confirm species affiliation. The extended quadruplex PCR phylo-group assignment method allowed the isolates to be classified into phylo-groups B1 (intestinal commensals), B2 and D (extraintestinal pathogens): 15 strains belonged to group B1 (55.6%), 9 to group B2 (33.3%), and 3 to group D (11.1%) (Table 1).

**Table 1:** Affiliation of the studied *E. coli* strains (*n* = 27) to phylo-groups

Phylo-group	Number of strains
Group B1	15
Group B2	9
Group D	3

**Fig. 1:**
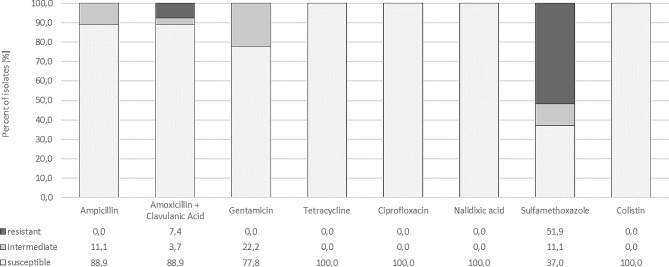
Sensitivity of *E. coli* strains (*n* = 27) to selected antimicrobials

Antibiotic resistance testing using the disk-diffusion method showed that 2 strains were resistant to amoxicillin with clavulanic acid (7.4%), and one was intermediate resistant (3.7%). Resistant to sulfamethoxazole were 14 strains (51.9%), and 3 were intermediate resistant (11.1%). To ampicillin, 3 strains were intermediate resistant (11.1%), and to gentamicin, 6 (22.2%). All tested strains (*n* = 27) were susceptible to: ciprofloxacin, tetracycline and nalidixic acid; of which 8 strains (29.6%) were sensitive to all tested antibiotics (Fig. 1). Furthermore, all isolates (100%) were susceptible to colistin in a broth microdilution test.

Molecular studies did not reveal the presence of any of the antibiotic resistance genes: *sul1, sul2, sul3, bla*_*TEM*_, *bla*_*CTX−M*_, *bla*_*SHV*_, *aac(6)-Ib-cr, tetA, tetB, and mcr-1, mcr-2, mcr-3, mcr-4, mcr-5.* The presence of 3 virulence genes was detected in the studied *E. coli* strains. The *ipr2* gene was present in 7 strains (25.9%), among which 6 of them also had the *vat* gene (22.2%). In one isolate, the *iucD* gene was detected (3.7%). The results were correlated with the phylo-group membership of the strains in question (Table 2).

**Table 2:** Occurrence of virulence genes and affiliation of studied *E. coli* strains (*n* = 27) to phylo-groups

Virulence gene	Number of strains	Phylo-group
*irp2*	7	B1
*irp2/vat*	6	B2
*iucD*	1	D

## Discussion

It is well known that increasing human interference in the environment leads to exchange of pathogens between humans and wildlife. There are still some unknowns regarding the transmission of *E. coli* itself, its genetic diversity, and the routes of infection between humans and wildlife [17]. Antimicrobial resistance among the microflora of free-living birds is a growing problem. This is also confirmed by studies conducted in Poland on *E. coli* strains present in wild birds: aquatic [18, 19], as well as terrestrial species [20]. Monitoring the increasing resistance of bacteria, which are intestinal commensals, is of particular importance because it can serve as an indicator of the level of environmental pollution and the accumulation of antimicrobial agents in ecosystems. It also provides a good reference when comparing antibiotic resistance in different animal groups and animal production sectors [21, 22]. It should be taken into account that bacteria that carry resistance genes can be isolated from slaughter animals and transmitted to humans, among other veterinary staff. In addition, microorganisms are under very strong pressure from the excessive use of bactericidal and bacteriostatic substances [21].

Nearly half (51.9%) of the strains were found to be resistant or intermediate resistant to sulfamethoxazole, but none of the three sulfonamide resistance genes tested - *sul1*, *sul2* or *sul3* - were present. On the other hand, Nowaczek et al. [20] reported a lower percentage of resistance for trimethoprim/sulfamethoxazole (34.3%) and confirmed the presence of the *sul2* and s*ul3* gene among strains resistant to this antimicrobial. In our results, the lack of sensitivity to this antibiotic may be due to the presence of another gene variant (*sul4*), or chromosomal resistance determined by a mutation in the dihydropteroate synthase (*folP*) enzyme gene [23–25]. Interestingly, in studies on *E. coli* in wild birds, sulfonamide resistance has been repeatedly demonstrated, but it was most often correlated with the presence of sulfonamide resistance genes [20, 22], which were not detected in the conducted study.

More than half (*n* = 15) of the *E. coli* isolates obtained from black grouse belong to phylo-group B1, indicating the dominance of non-pathogenic strains in the study population, while the rest were classified into groups B2 (*n* = 9) and D (*n* = 3), comprising potentially virulent strains [26]. The results obtained for the phylo-groups correlate with the presence of the virulence genes shown in the study − 6 strains belonging to group B2 had the virulence genes *irp2* and *vat*, *and 1* strain from group D had the *iucD* gene. Despite the assumption that phylo-group B1 contains non-pathogenic strains, in this study 1 of the strains possessed the virulence gene *irp2*. Differences in pathogenicity within a single phylo-group have also been observed in previous studies on APEC [27]. In addition, a similar result was obtained in a study by Nowaczek et al. [20] on wild birds in Poland, where virulence genes were detected in strains belonging to phylo-group B1, however, their frequency was significantly lower than in groups B2 and D.

The virulence genes selected for analysis are responsible for the pathogenicity of APEC isolates that cause colibacteriosis in poultry flocks [28]. There are also hypotheses, supported by molecular studies, comparing APEC strains with ExPEC (Extraintestinal Pathogenic *Escherichia coli*). These experiments indicate that a group of extraintestinal *E. coli* may have zoonotic potential and cause infections in humans [4]. On the other hand, there is also the possibility of reverse transmission from humans to animals [29].

The most frequently isolated virulence gene was the *irp2* gene, responsible for the production of an iron repressible-protein, and the *vat* gene, encoding a vacuolating autotransporter toxin. In studies on wild birds in Poland and the Netherlands, the *irp2* gene was also shown to be one of the most common virulence genes in *E. coli* [19, 20]. Similar studies were performed in a group of wild birds, where the frequent occurrence of virulence genes *iss* and, less frequently, *tsh* (also genes characteristic of APEC strains), were demonstrated [30]. In the aforementioned work, an examination of eagle and hawk feces also showed the presence of *E. coli* strains with significant antibiotic resistance. Whereas, in the feces of migratory birds in Bangladesh, *E. coli* strains possessing *iucD* and *papC* genes have been identified, which are also associated with APEC [31]. In the same study, these *E. coli* strains were found to be significantly resistant to ciprofloxacin, tetracycline, or ampicillin. The reports of researchers who obtain these results indicate that there is a ground for further research on other groups of wild birds in different regions of the world.

This study also showed that feces samples collected from black grouses can be fibrous (hard and cylindrical) or soft (without form and semi-liquid). In the winter months, feces with a hard and fibrous structure predominate. Furthermore, it should be noted that significantly fewer strains of *E. coli* and other bacterial species have been cultured from samples with this structure under laboratory conditions. This decrease in the amount and diversity of the intestinal microbiome may be related to the black grouse diet, which in winter is based on the needles and buds of coniferous trees [32, 33]. Although the winter months facilitate quick and aseptic sampling for microbiological tests, it is much more difficult to isolate bacteria from the fecal samples of these birds.

The insufficient scientific data in the literature on the gastrointestinal microbiome of black grouse makes it impossible to conduct a comparative analysis of their different populations. Further research is required to determine the composition and interactions between the bacterial species that form the physiological flora of these birds. Additionally, the assessment seasonal changes in intestinal microbes composition depending on changes in wild birds diet should be also performed [32]. This knowledge will contribute to protecting the populations of bird species and improving their welfare.

## Conclusions

Although there is a possibility that wild birds carry resistant bacteria that are passed on to them by humans or other animals, our study demonstrated the sensitivity of *E. coli* strains to most selected antibiotics, including the most commonly used in human medicine. First, our results present the low degree of antibacterial substances transmission into the natural habitat of black grouse. Second, the strains isolated from black grouse are unlikely to be of anthropogenic origin. It is possibly due to the Karkonosze National Park’s activities about the preservation and restoration of black grouse’s habitat, among other things, through the control of forestry works and tourist traffic.

## Methods

### **Isolation of* E. coli* strains**

The study included a total of 197 black grouse fecal samples, collected in the Karkonosze National Park of approximately 25 km^2^ area and 1000–1250 m above sea level, from places where the birds spent the night, during the winter months of 2017 and 2018. The collected feces samples were placed in sterile containers and immediately transported in an isothermal box with freezing inserts to the “Epi-Vet” Veterinary Diagnostic Laboratory. For microbiological examination, 1 g of fresh feces was incubated for 16–20 h at 37^o^C in BHI (Brain Heart Infusion broth) (Oxoid, Hampshire, United Kingdom). The suspension was then inoculated onto McConkey’s agar (Oxoid, Hampshire, United Kingdom) and incubated for 18 h at 37^o^C. The single, lactose-positive colonies characteristic of *E. coli* were transferred to nutrient agar medium (Merck, Darmstadt, Germany). The isolates were preserved for further testing at -70^o^C, using the Microbank system (Pro-lab Diagnostics, Richmond Hill, Canada). Furthermore, isolated bacteria were identified as *E. coli* by observing their cultural characteristics, morphology by Gram’s stain, oxidase test, EMB (eosin methylene blue) agar and motility test. Presumptive *E. coli* strains were selected for further PCR analysis.

### Antimicrobial-susceptibility testing

All *E. coli* isolates were subjected to drug resistance testing using the disk diffusion method and the broth microdilution technique for colistin. *Escherichia coli* ATCC 25,922 was used as an internal control for both the disk diffusion method and the establishment of the minimum inhibitory concentration (MIC) of colistin. The bacteria were inoculated on MacConkey agar (Oxoid, Hampshire, United Kingdom) and incubated for 18 h at 37 °C. Subsequently, bacteria suspensions were prepared in 0.9% NaCl with an optical density of 0.5 McFarland. For the disc diffusion method, the bacteria suspensions were spread onto Mueller-Hinton agar (Oxoid, Hampshire, United Kingdom) using a sterile swab. Susceptibility to the following antimicrobial agents was determined: amoxicillin with clavulanic acid (20/10 µg) (Bio-Rad, California, USA), ciprofloxacin (5 µg), tetracycline (30 µg), sulfamethoxazole (25 µg), gentamicin (10 µg), ampicillin (10 µg) and nalidixic acid (30 µg) (Oxoid, Hampshire, United Kingdom). The inhibition zones were identified and visually interpreted. The minimum inhibitory concentration sensitivities for colistin were determined using a broth microdilution technique with a ComASP Colistin panel (Liofilchem, Via Scozia, Italy) following the suggested manufacturer protocol using Mueller-Hinton broth. The antimicrobial resistance phenotyping of the isolates was performed and interpreted according to the Clinical and Laboratory Standards Institute (CLSI) M100 ED23; 2023 [34].

### Molecular analysis

The isolation of genomic DNA was performed using a Genomic Mini kit (A&A Biotechnology, Gdańsk, Poland) according to the instructions provided by the producer. The isolated genetic material was stored at -70^o^C for further studies.

The species verification of all isolates was carried out using a PCR reaction based on the amplification of the *phoA* and *16 S* rRNA genes [35, 36]. The *E. coli* strains were then subjected to the extended quadruplex phylo-typing analysis according to the methods described by Clermont et al. [26]. Additionally, the presence of virulence genes was tested: heat-stable enterotoxin (*astA*), increased serum survival protein (*iss*), iron repressible-protein (*irp2*), pyelonephritis-associated pili (*papC*), iron acquisition-related factors aerobactin (*iucD*), temperature-sensitive hemagglutinin (*tsh*), vacuolating autotransporter toxin (*vat*), gene from colicin V plasmid operon genes (*cvi/cvaC*) and verocytotoxigenic cytotoxin (*stx2f*) [28, 37].

Based on the susceptibility results to selected antimicrobial agents, the presence of the following resistance genes was checked: sulfonamides (*sul1*, *sul2*, *sul3*), beta-lactams (*bla*_*TEM*_, *bla*_*CTX−M*_, *bla*_*SHV*_), aminoglycosides (*acc(6’)-Ib-cr*), tetracyclines (*tetA*, *tetB*) and colistin (*mcr-1*, *mcr-2*, *mcr-3*, *mcr-4*, *mcr-5*). Reactions were carried out according to previous studies [38–40]. The primers and their annealing temperatures are listed in Additional file 1. The positive control in individual PCR reactions was the DNA templates confirmed by Sanger sequencing.

### Electronic supplementary material

Below is the link to the electronic supplementary material.

Additional file 1. Primer sequences, their annealing temperatures, and the size of PCR reaction products.

## Data Availability

The datasets used and analysed during the current study are included in the article.

## Abbreviations

APEC: Avian Pathogenic *Escherichia coli*
BHI: Brain Heart Infusion broth
CLSI: Clinical and Laboratory Standards Institute
*E. coli*: *Escherichia coli*
ExPEC: Extraintestinal Pathogenic *Escherichia coli*
NMEC: Neonatal Meningitis *Escherichia coli*
UPEC: Uropathogenic *Escherichia coli*
