# Supplementary material for: Occurrence and molecular characterization of Escherichia coli strains isolated from black grouse (Lyrurus tetrix) from the Karkonosze National Park in Poland
Source: BMC Vet Res. 2024 Jan 31;20:38. doi: 10.1186/s12917-024-03886-3 (PMC10829180; doi:10.1186/s12917-024-03886-3)
Supplement: Supplementary file 1 — Additional file 1. Primer sequences, their annealing temperatures, and the size of PCR reaction products. [file 12917_2024_3886_MOESM1_ESM.docx]

Table S1. Primer sequences, their annealing temperatures and the size of PCR reaction products.

| **Gene** | | **Primer sequence** | **Annealing temp. [C°]** | **Size of product (bp)** | **Reference** |
| --- | --- | --- | --- | --- | --- |
| *phoA* | | 5’- GCACTCTTACCGTTACTGTTTACCCC-3’  5’- TTGCAGGAAAAAGCCTTTCTCATTTT-3’ | 58 | 1001 | [35] |
| *16s rRNA* | | 5’- GGGAGTAAAGTTAATACCTTTGCTC -3’  5’- TTCCCGAAGGCACATTCT -3’ | 60 | 585 | [36] |
| *chuA* | | 5’- ATGGTACCGGACGAACCAAC -3’  5’- TGCCGCCAGTACCAAAGACA -3’ | 59 | 288 | [26] |
| *yjaA* | | 5′-CAAACGTGAAGTGTCAGGAG-3′  5′-AATGCGTTCCTCAACCTGTG-3′ |  | 211 |  |
| *TspE4.C2* | | 5′-CACTATTCGTAAGGTCATCC-3′  5′-AGTTTATCGCTGCGGGTCGC-3′ |  | 152 |  |
| *arpA* | | 5′-AACGCTATTCGCCAGCTTGC-3′  5′-TCTCCCCATACCGTACGCTA-3′ |  | 400 |  |
| *arpA-C* | | 5′-GATTCCATCTTGTCAAAATATGCC-3′  5′-GAAAAGAAAAAGAATTCCCAAGAG-3′ | 57 | 301 |  |
| *trpA-C* | | 5′-AGTTTTATGCCCAGTGCGAG-3′  5′-TCTGCGCCGGTCACGCCC-3′ | 59 | 219 |  |
| *trpA* | | 5′-CGGCGATAAAGACATCTTCAC-3′  5′-GCAACGCGGCCTGGCGGAAG-3′ | 59 | 489 |  |
| *astA* | | 5’-TGCCATCAACACAGTATATCC-3’  5’-TCAGGTCGCGAGTGACGGC-3’ | 58 | 116 | [37] |
| *iss* | | 5’-ATCACATAGGATTCTGCCG-3’  5’-CAGCGGAGTATAGATGCCA-3’ |  | 309 |  |
| *irp2* | | 5’-AAGGATTCGCTGTTACCGGAC-3’  5’-AACTCCTGATACAGGTGGC-3’ |  | 413 |  |
| *papC* | | 5’-TGATATCACGCAGTCAGTAGC-3’  5’-CCGGCCATATTCACATAA-3’ | 60 | 501 | [28] |
| *iucD* | | 5’-ACAAAAAGTTCTATCGCTTCC-3’  5’-CCTGATCCAGATGATGCTC-3’ |  | 714 |  |
| *tsh* | | 5’-ACTATTCTCTGCAGGAAGTC-3’  5’-CTTCCGATGTTCTGAACGT-3’ | 57 | 824 |  |
| *vat* | | 5’-TCCTGGGACATAATGGTCAG-3’  5’-GTGTCAGAACGGAATTGT-3’ |  | 981 |  |
| *cva A/B*  *cvi cvaC* | | 5’-TGGTAGAATGTGCCAGAGCAAG-3’  5’-GAGCTGTTTGTAGCGAAGCC-3’ |  | 1181 |  |
| *stx2f* | | 5’-AGATTGGGCGTCATTCACTGGTTG-3’  5’-TACTTTAATGGCCGCCCTGTCTCC-3’ |  | 428 |  |
| *mcr-1* | | 5’-AGTCCGTTTGTTCTTGTGGC-3’  5’-AGATCCTTGGTCTCGGCTTG-3’ | 63 | 320 | [39] |
| *mcr-2* | | 5’-CAAGTGTGTTGGTCGCAGTT-3’  5’-TCTAGCCCGACAAGCATACC -3’ |  | 715 |  |
| *mcr-3* | | 5’-AAATAAAAATTGTTCCGCTTATG -3’  5’-AATGGAGATCCCCGTTTTT-3’ |  | 929 |  |
| *mcr-4* | | 5’-TCACTTTCATCACTGCGTTG -3’  5’-TTGGTCCATGACTACCAATG-3’ |  | 1116 |  |
| *mcr-5* | | 5’-ATGCGGTTGTCTGCATTTATC -3’  5’-TCATTGTGGTTGTCCTTTTCTG-3’ |  | 1644 |  |
| *bla_CTX-M_* | | 5’-AGTGAAAGCGAACCGAATC-3’  5’-CTGTCACCAATGCTTTACC-3’ | 55 | 365 | [40] |
| *bla_TEM_* | | 5’-CAGAAACGCTGGTGAAAGTA-3’  5’-ACTCCCCGTCGTGTAGATAA-3’ |  | 719 |  |
| *bla_SHV_* | | 5’- ATGCGTATATTCGCCTGTG-3’  5’-CCTCATTCAGTTCCGTTTCC-3’ |  | 502 |  |
| *acc(6′)-Ib-cr* | | 5’-CGATCTCATATCGTCGAGTGTT-3’  5’-TTAGGCATCACTGCGTGTTC-3’ | 50 | 447 | [38] |
|  | | | | |  |
| *tetA* | | 5’-GGCCTCAATTTCCTGACG-3’  5’-AAGCAGGATGTAGCCTGTGC-3’ | 57 | 372 | [40] |
| *tetB* | | 5’-GAGACGCAATCGAATTCGG-3’  5’-GAGACGCAATCGAATTCGG-3’ |  | 228 |  |
| *sul1* | | 5’-GTGACGGTGTTCGGCATTCT-3’  5’-TCCGAGAAGGTGATTGCGCT-3’ | 68 | 779 |  |
| sul2 | | 5’-CGGCATCGTCAACATAACCT-3’  5’-TGTGCGGATGAAGTCAGCTC-3’ | 66 | 721 |  |
| *sul3* | | 5’-GAGCAAGATTTTTGGAATCG-3’  5’-CATCTGCAGCTAACCTAGGGCTTTGGA-3’ | 51 | 880 |  |
